# A Young Adult Presenting With an Abdominal Aneurysm Due to Cystic Medial Necrosis of the Aorta

**DOI:** 10.7759/cureus.41966

**Published:** 2023-07-16

**Authors:** Venkata Vineeth Vaddavalli, Sasi Karnati, Kishore Abuji, Ajay Savlania

**Affiliations:** 1 Department of General Surgery, Postgraduate Institute of Medical Education and Research, Chandigarh, IND

**Keywords:** trauma, saccular aneurysm, aneurysm repair, abdominal aortic aneurysm, cystic medial necrosis

## Abstract

Cystic medial necrosis is a disorder of large arteries, particularly the thoracic aorta, characterized by an accumulation of a basophilic ground substance in the media with cyst-like lesions. A male in his late 20s was brought to our trauma bay after he met with a road traffic accident with a complaint of abdominal pain. Clinical examination revealed tenderness in the left lumbar region. The contrast-enhanced computerized tomography revealed an aneurysm of size 11×9.6×9.2 cm in the left lateral aspect of the abdominal aorta at the origin of the left renal artery. Intraoperatively, an aneurysm of size 10×10 cm from the juxta renal abdominal aorta was identified, and aortic rent was repaired with a polyester graft. The tissue was sent for histopathology, which showed complicated atherosclerosis with cystic medial degeneration and aneurysmal rupture of the abdominal aorta containing thrombus. The patient had an uneventful postoperative course and is doing well without any complaints at a two-year follow-up.

## Introduction

The prevalence of abdominal aortic aneurysm (AAA) in the United States is estimated at around two to eight percent, increasing with age with a male predisposition [1]. The aneurysms are usually secondary to atherosclerosis, trauma, developmental causes, or due to congenital anomalies and infections. Cystic medial degeneration or necrosis as a cause of an AAA is rare, with only a single case reported in the literature to the best of the literature search [2]. Cystic medial necrosis (CMN) is a disorder of large arteries, particularly the thoracic aorta, characterized by an accumulation of a basophilic ground substance in the media with cyst-like lesions [3]. It is known to occur mainly in connective tissue diseases such as Marfan's syndrome, Ehlers-Danlos syndrome, and annuloaortic ectasia because of degenerative changes in the aortic wall [4-6]. We report a case of ruptured AAA due to CMN in a male in his late 20s, who is non-hypertensive without any history of connective tissue disorders.

## Case presentation

**Figure 1:**
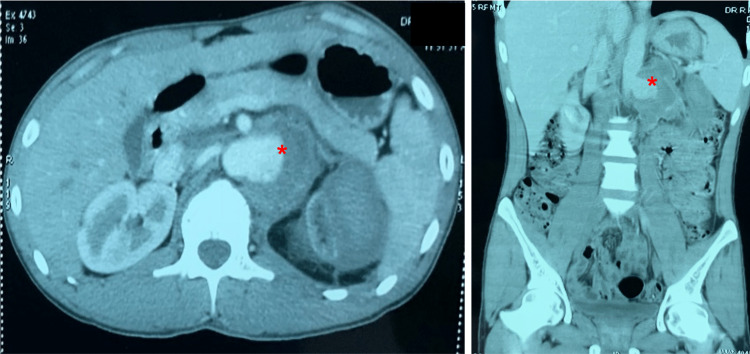
Axial and coronal sections showing the aneurysm (marked by an asterisk)

A young gentleman who had suffered a road traffic accident one week ago presented with complaints of abdominal pain. The patient was conscious, well-oriented, and hemodynamically stable. On palpation, mild tenderness was present in the left lumbar region. Hematological and biochemical parameters were within normal limits. His height was 168cm, and his weight was 54 kilograms, with a body mass index of 19.1 kg/m^2^. Abdominal ultrasonography revealed a saccular outpouching from the left lateral aspect of the abdominal aorta inferior to the superior mesenteric artery at the level of the left renal artery with a partial thrombus. The contrast-enhanced computerized tomography revealed an aneurysm of size 11×9.6×9.2 cm in the left lateral aspect of the abdominal aorta at the origin of the left renal artery (Figure 1).

**Figure 2:**
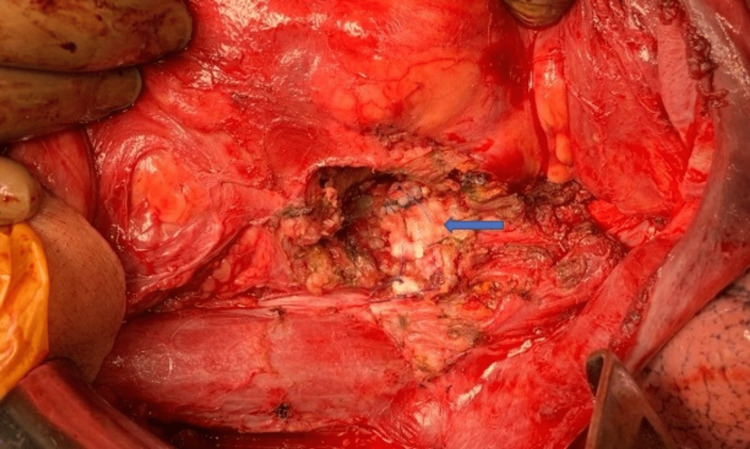
Aortic rent repair done using the polyester patch (solid arrow)

There was no free fluid in the abdomen, and soft tissue planes were well-defined without any fat stranding surrounding the aneurysm. The patient was evaluated and prepared for open surgical repair. After optimizing the patient, he was taken up for open surgical repair. A midline incision was made over the abdomen, and the retroperitoneum was exposed. Intraoperatively, there was no hematoma or signs of acute inflammation. A saccular aneurysm measuring 10×10 cm from the juxta renal abdominal aorta was found, and the left kidney was atrophic. After opening the sac and removal of the thrombus, the aortic rent was found, and it was repaired using a polyester patch (Figure 2). The postoperative period was uneventful, and the patient was discharged on postoperative day seven. He is doing well without any complaints at the two-year follow-up. The follow-up computed tomography angiography showed a mild bulge at the polyester patch site, and the rest of the thoracic and abdominal aorta revealed no abnormalities.

**Figure 3:**
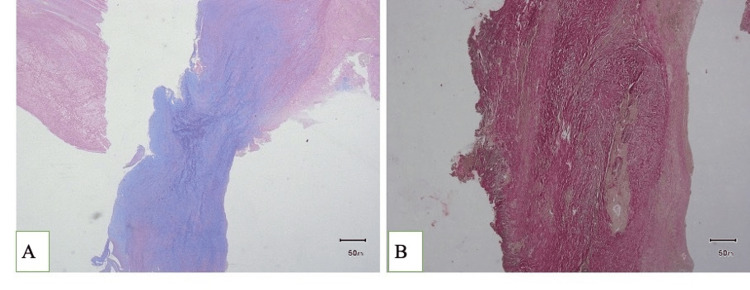
A) PAS-Ab stain showing myxoid degeneration and B) EVG stain showing disrupted elastic lamina PAS-Ab, periodic acid Schiff's and Alcian blue; EVG, elastic Von Geison

On histopathological examination, sections from the abdominal aortic wall showed extensive fibrosis with fibroblast proliferation, dense lymphoplasmacytic inflammatory infiltrates admixed with neutrophils and focal lymphoid aggregates in the media. In addition, myxoid degeneration was also noted on periodic acid Schiff's and Alcian blue (PAS-Ab) stain (Figure 3A). Disrupted elastic lamina was noted on the elastic Von Geison (EVG) stain (Figure 3B). Overall features were suggestive of complicated atherosclerosis with aneurysmal rupture and cystic medial degeneration. 

## Discussion

Babes and Mironescu described the CMN in aortic dissection in 1910 [7]. In 1928, Gsell and Erdheimin proposed the concept of idiopathic CMN related to aortic aneurysm, dissection, and rupture in Marfan's syndrome [8]. The prevalence of CMN is estimated to be six percent in patients with thoracic aortic aneurysms. The incidence of cystic medial degeneration increases progressively with age, reaching up to 60% and 64% in the seventh and eighth decade, respectively [9]. In our case, the patient was young and with no features of hypertension and connective tissue disorders. The aortic aneurysm caused by cystic medial degeneration is usually limited to ascending aorta. The difference in composition between the ascending and descending aorta might be responsible for the difference in the etiology of aneurysms that develop in these areas. Ascending aorta has a greater concentration of elastic fibers and is more compliant than the descending aorta. Thus, most thoracic aortic aneurysms are associated with degenerative changes in the elastic media. In contrast, most descending aortic aneurysms and AAAs are associated with atherosclerosis [10]. This denotes that CMN in the development of AAA is an exceedingly rare feature, with only one case report in the literature.

CMN is not the cause but a common pathological finding secondary to primary disorder, like fibrillin deficiency or advanced apoptosis. CMN is a common finding in elastic arterial specimens, and the difference between normal and abnormal is the amount of ground substance in the media [9]. In patients without Marfan's syndrome, CMN occurs more frequently in geriatric and hypertensive individuals [11]. In our case, the patient was young, non-hypertensive, and did not have a known case of connective tissue disorder. To the best of the literature search, only one case was reported by JS Munn et al. in a middle-aged woman without Marfan's syndrome. The exact incidence or prevalence due to cystic medial degeneration is unknown. When we had a retrospection into our case, initially, we suspected that the patient had a pseudoaneurysm of the abdominal aorta due to the antecedent history of trauma, and the initial ultrasound demonstrated a saccular outpouching from the abdominal aorta. On the cross-sectional imaging, there was an absence of free fluid in the abdomen and no fat stranding surrounding the aneurysmal sac. Additionally, the patient had no hematoma or signs of acute inflammation intraoperatively, and there was an organized thrombus in the sac with an atrophic left kidney, which suggests chronic pathology. Finally, with supportive evidence of CMN and an aneurysm wall showing all three layers in the histopathological report, we are inclined toward the diagnosis of a saccular aneurysm of the abdominal aorta than a traumatic pseudoaneurysm. As the CMN predisposes the patient to the aneurysms of ascending and descending thoracic aorta, we plan to keep the patient on a regular follow-up. 

## Conclusions

CMN as a cause of AAA is exceedingly rare in young patients. The literature regarding the characteristics of this entity is lacking. Hence, we must call on vascular surgeons worldwide to report more such cases to comment on the epidemiology and clinical presentation. However, moving toward the endovascular treatment for aortic aneurysms might be a roadblock to learn about the pathological causes of the aneurysms.
